# Association of Hemoglobin Glycation Index With Contrast-Induced Acute Kidney Injury in Patients Undergoing Coronary Angiography: A Retrospective Study

**DOI:** 10.3389/fphys.2022.870694

**Published:** 2022-05-20

**Authors:** Zhezhe Chen, Duanbin Li, Maoning Lin, Hangpan Jiang, Tian Xu, Yu Shan, Guosheng Fu, Min Wang, Wenbin Zhang

**Affiliations:** ^1^ Department of Cardiology, Sir Run Run Shaw Hospital, College of Medicine, Zhejiang University, Hangzhou, China; ^2^ Key Laboratory of Cardiovascular Intervention and Regenerative Medicine of Zhejiang Province, Hangzhou, China; ^3^ Department of Cardiology, The Fourth Affiliated Hospital, College of Medicine, Zhejiang University, Yiwu, China

**Keywords:** hemoglobin glycation index, contrast-induced acute kidney injury, coronary angiography, percutaneous coronary intervention, coronary artery disease

## Abstract

**Background:** The hemoglobin glycation index (HGI) quantifies interindividual variation in glycation and is positively associated with cardiovascular diseases. However, the association between HGI and contrast-induced acute kidney injury (CI-AKI) remains unclear. Therefore, this study aimed to assess the association of HGI with CI-AKI.

**Methods:** In this observational study, a total of 3,142 patients undergoing coronary angiography (CAG) or percutaneous coronary intervention (PCI) were included. The HGI was calculated as the difference between the measured glycated hemoglobin (HbA1c) and predicted HbA1c. CI-AKI was defined as an increase of either 25% or 0.5 mg/dl (44.2 μmol/L) in the serum creatinine (SCr) level within 72 h following the exposure to contrast medium. Piecewise linear regression analysis was conducted to testify the association of HGI with the proportion of SCr elevation. Modified Poisson’s regression analysis was performed to determine the association between HGI and CI-AKI. Exploratory analysis was also performed according to the stratification of HbA1c levels.

**Results:** Among 3,142 patients, the average age was 66.9 years and 483 of them (15.4%) suffered CI-AKI. Piecewise linear regression analysis demonstrated the linear association of HGI with the proportion of SCr elevation on both positive and negative sides of HGI [HGI <0: *β* = −9.537, 95% CI (−12.057 to −7.017), *p* < 0.001; HGI ≥0: *β* = 1.655, 95% CI (0.125 to 3.186), *p* = 0.034]. Modified Poisson’s regression analysis showed that the higher absolute value of HGI was strongly associated with higher incidence of CI-AKI [(<−1.0 vs*.* −0.2 to 0.2): aRR = 1.897, 95% CI [1.467 to 2.452], *p* < 0.001 (≥1.0 vs*.* −0.2 to 0.2): aRR = 1.545, 95% CI (1.171 to 2.037), *p* = 0.002]. Furthermore, the results in exploratory analysis showed that such association still remained irrespective of HbA1c levels.

**Conclusion:** The higher absolute value of HGI was strongly associated with higher incidence of CI-AKI in patients undergoing CAG and PCI.

## Introduction

Coronary artery disease (CAD) is a leading killer of human beings worldwide (Malakar et al., 2019). Recently, coronary angiography (CAG) and percutaneous coronary intervention (PCI) have been used as essential methods for diagnosis and treatment, dramatically improving the prognosis (Bhatt, 2018). However, the risk of complications of CAG or PCI cannot be ignored and remains a tricky problem (Giannini et al., 2018).

Contrast-induced acute kidney injury (CI-AKI) is an acute loss of renal function following contrast exposure, which ranks as the third leading cause of iatrogenic acute renal failure (McCullough et al., 2016). According to the European Society of Urogenital Radiology (ESUR), the definition for CI-AKI is based on a serum creatinine (SCr) elevation of more than 44 μmol/L (0.5 mg/dl) or 25% from baseline within 72 h in the absence of an alternative cause (Stacul et al., 2011; Fähling et al., 2017). The incidence of CI-AKI ranges from 5 to 25% and varies substantially due to different risk factors (Mehran et al., 2004; Shaker et al., 2010). It has been reported that both procedure- and patient-related risk factors can impact the initiation and progression of CI-AKI, including the volume of contrast agents, pre-existing kidney failure, diabetes, and hemodynamic instability (Azzalini et al., 2016). Our previous studies also proved that the severity of heart failure and the absence of statin application before admission were both independent predictors for CI-AKI (Lin et al., 2021; Xu et al., 2021). Considering that CI-AKI can increase the risk of mortality, it is important to identify risk factors for early detection and prevention of the incidence of CI-AKI (Legnazzi et al., 2020).

Diabetes and pre-diabetes have served as classic risk factors for CI-AKI (Toprak et al., 2007). Moreover, current evidence shows that patients with poor control of blood glucose have an increased risk of CI-AKI (Calvin et al., 2010). Therefore, indicators that can quantify patient recent glycemic control may provide more value to CI-AKI. Elevated glycated hemoglobin (HbA1c), indicating an increased average level of blood glucose and representing poor blood glucose control in the past 2–3 months, was found to be tightly associated with a greater risk for CI-AKI (Ding et al., 2013). Moreover, preoperative fasting blood glucose (FBG) levels have been proven to be independently associated with CI-AKI (Zhang et al., 2022). However, limitations still exist such as HbA1c cannot reflect fluctuations of glucose control flexibly and FBG is easily affected by stress and recent diet (Zhang et al., 2021).

Defined as the difference between the observed value of HbA1c and the predicted HbA1c based on plasma glucose levels, the hemoglobin glycation index (HGI) represents the degree of nonenzymatic hemoglobin glycation (Hempe et al., 2002). As a combination of HbA1c and FBG, HGI is now considered a more stable indicator that maintains consistency within a wide range of blood glucose concentration, over time (McCarter et al., 2006; Soros et al., 2010). It is now well-established from a variety of research studies that HGI was closely associated with the progression of multiple diseases and could be a great predictor of liver diseases and cardiovascular diseases (Fiorentino et al., 2017a; van Steen et al., 2017; Kim et al., 2018). However, the association between HGI and CI-AKI has not yet been fully understood. Therefore, this study was conducted to demonstrate the association of HGI with the incidence of CI-AKI in patients undergoing CAG or PCI.

## Methods

### Study Population

**FIGURE 1:**
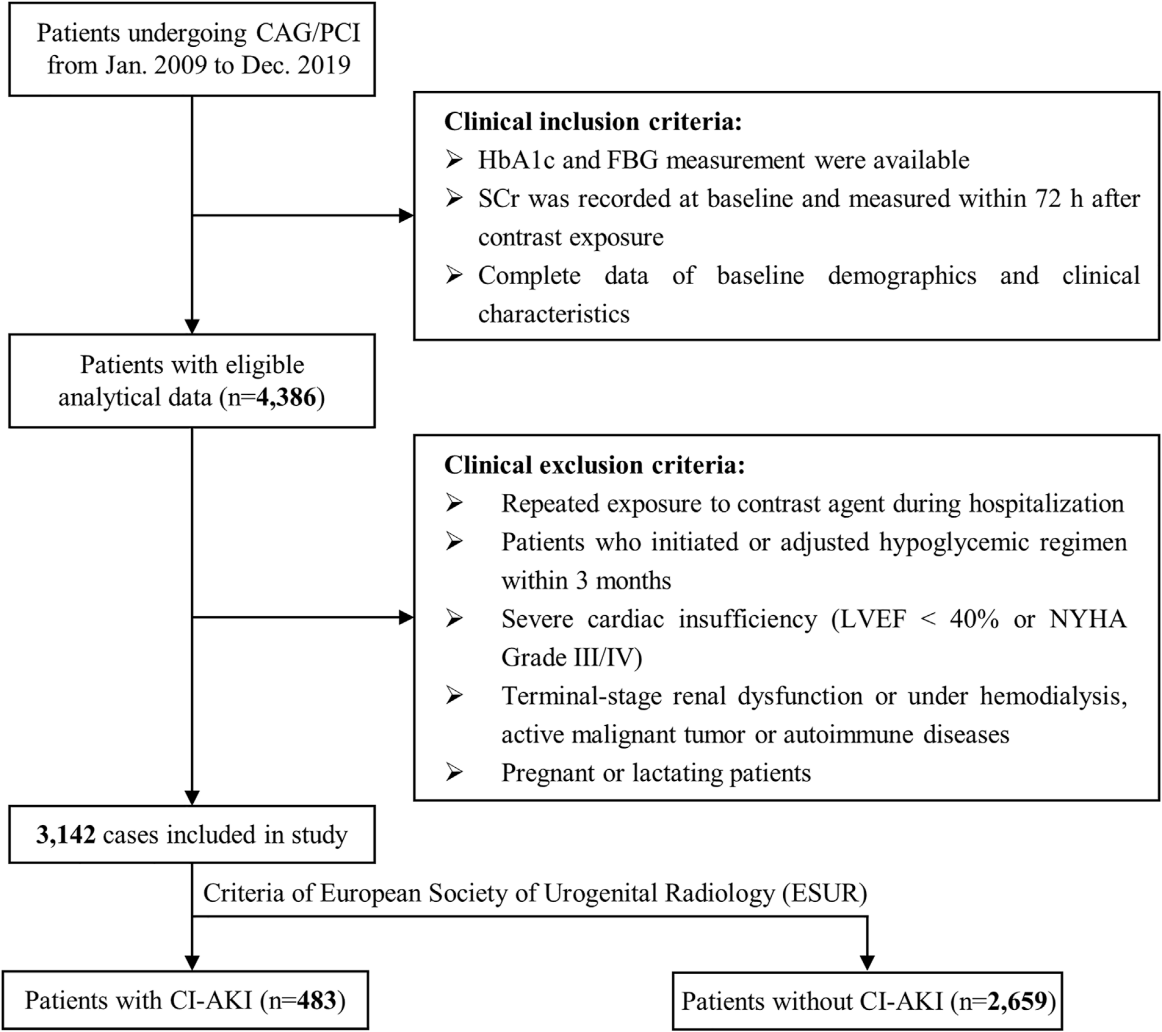
Inclusion and exclusion flowchart. The flowchart described the inclusion and exclusion criteria of study participants. CAG indicates coronary angiography; CI-AKI, contrast-induced acute kidney injury; FBG, fasting blood glucose; HbA1c, glycated hemoglobin; LVEF, left ventricular ejection fractions; NYHA, New York Heart Association; PCI, percutaneous coronary intervention; SCr, serum creatinine.

A retrospective observational study was conducted, and individuals who underwent CAG or PCI from January 2009 to December 2019 in Sir Run Run Shaw Hospital and its medical consortium hospitals were recruited. The flowchart of patient screening is shown in Figure 1. Inclusion criteria were as follows: 1) available data of HbA1c and fasting blood glucose (FBG); 2) documented SCr levels at baseline and within a 72 h timeframe after contrast exposure; and 3) complete data of baseline characteristics. Exclusion criteria were as follows: 1) patients with repeated contrast exposure in addition to this procedure; 2) patients who started or adjusted the hypoglycemic regimen within 3 months; 3) patients with severe cardiac insufficiency (left ventricular ejection fraction (LVEF) < 40% or New York Heart Association (NYHA) Grade III/IV); 4) patients with existing end-stage renal dysfunction or undergoing hemodialysis, active malignant tumor, or autoimmune diseases; and 5) pregnant or lactating mothers. The implementation of this study was reviewed and approved by the Ethics Review Committee of Sir Run Run Shaw Hospital (NO. 20201217-36). This study also followed the Strengthening the Reporting of Observational Studies in Epidemiology (STROBE) (Vandenbroucke et al., 2007).

### Sample Size Calculation

Sample size calculation was based on the thumb rule that the sample size should be 5, 10, or 20 times the number of variables (Norman et al., 2012). Twenty positive events for each variable would meet the most stringent rule of thumb. In this study, nine variables were finally included and, thus, 180 positive patients were needed. In previous studies, the incidence of CI-AKI ranges from 5 to 25%, and the median value of 15% was chosen to be the estimated incidence of CI-AKI. Therefore, at least 1200 patients were required. This observational study enrolled 3,142 patients and 483 with CI-AKI, which was far more than the required population.

### Quality Assessment

The validated Newcastle–Ottawa Scale (NOS) was applied to assess the quality of the study (Wells et al., 2013). Briefly, the scale comprises population selection, comparability of groups, and ascertainment of exposure and outcome. The total score ranges from 0 to 9. A score greater than or equal to 7 is considered to be high quality. Two reviewers assessed the study quality independently and any disagreement was adjudicated by a third reviewer.

### Definitions

**FIGURE 2:**
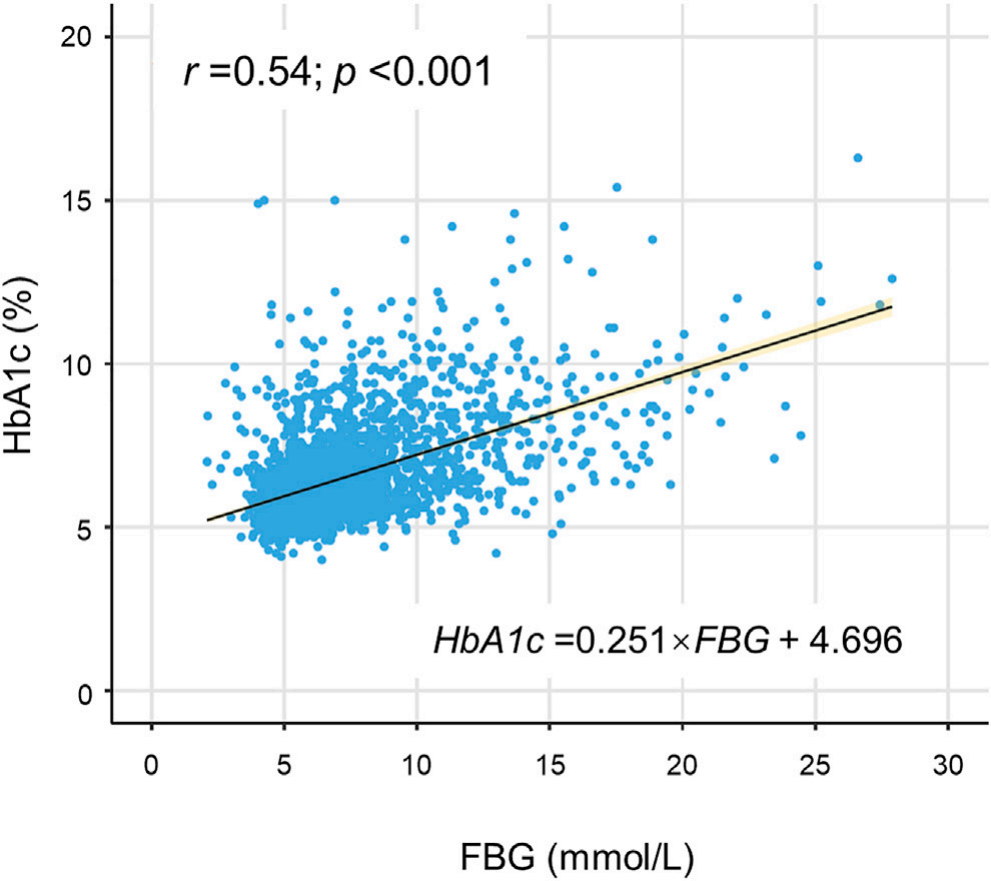
Correlation of FBG with HbA1c. A statistically significant positive association was shown between FBG and HbA1c in the scatterplot. The solid line was the linear regression fitted line, while the yellow shadow around the line indicated 95% confidence interval of the regression. FBG indicates fasting blood glucose; HbA1c, glycated hemoglobin.

HGI was defined as the difference between the measured HbA1c level and predicted HbA1c level (HGI = measured HbA1c - predicted HbA1c) (Hempe et al., 2002). Predicted HbA1c was calculated in each measured FBG value according to the equation derived from linear regression between HbA1c and FBG levels (predicted HbA1c = 0.251×FBG+4.696, *r* = 0.54, *p* < 0.001) (Figure 2). According to the positive or negative value, the HGI was divided into two pieces with 0 as the boundary. Moreover, the HGI was further categorized into seven groups with equal interval (per 0.4).

The baseline SCr was routinely tested on patient admission and the postoperative SCr was measured within 72 h after CAG or PCI. According to the European Society of Urogenital Radiology (ESUR), the CI-AKI was defined as an increase of postoperative SCr concentration above 25% of the baseline measurement or the absolute value over 0.5 mg/dl (Stacul et al., 2011; Fähling et al., 2017).

### Data Collection

Demographic information and clinical data of all subjects were extracted from the Hospital Information System (HIS). Baseline characteristics were queried and documented in all patients at hospital admission, including age, gender, body mass index (BMI), previous medical history, and recent medication history. Blood samples were collected after admission for blood routine and biochemical tests to obtain the HbA1c level and other laboratory measurements. FBG was detected in the morning after at least 8 h of fasting. Detailed information on the characteristics of CAG or PCI was also recorded after the procedure. The baseline SCr concentration was documented from the result of biochemical tests on admission. The postoperative SCr concentration was measured at least three times within 72 h after exposure of the contrast medium and the highest value was recorded.

### Statistical Analyses

Continuous variables were presented as mean ± standard deviation (SD) if normally distributed or median (interquartile range) if not. Categorical variables were expressed as numbers and proportions. Baseline comparability was assessed using Student’s *t*-test for normally distributed continuous variables or Mann–Whitney *U* test if not. Chi-square test or Fisher’s exact test were used to examine the baseline comparability for categorical variables as appropriate.

Spearman’s rank-order correlation analysis was performed to explore the association between FBG and HbA1c, and the linear regression model was fitted to build a regression equation for calculating predicted HbA1c. Piecewise linear regression analysis was performed to observe the linear trend on both positive and negative sides of HGI (HGI <0 and HGI ≥0). The multivariable regression analysis adjusted the potential significant predictors as covariates, including age (Marenzi et al., 2004), gender (Sidhu et al., 2008), hypertension (Lun et al., 2021), estimated glomerular filtration rate (eGFR) (McCullough et al., 2016), exposure volume of contrast agent (Marenzi et al., 2004), administration of statins (Li et al., 2016), ACEI/ARB (Ma et al., 2020), and diuretics (Yuan et al., 2017). The restricted cubic spline (RCS) curve was plotted to evaluate the association between the HGI and CI-AKI. Then, the HGI was categorized into seven groups with equal interval, and modified Poisson’s regression analysis was carried out to present the adjusted relative risk (aRR) of the HGI for CI-AKI. The aRR was also adjusted for the same covariates mentioned above. Finally, exploratory analysis was conducted in subgroups according to HbA1c levels (<6.0% or ≥6.0%) using modified Poisson’s regression model with full adjustment.

All statistical tests were 2-tailed and significance levels were set at the level of 5%. Data management and statistical analysis were performed using SPSS software (version 25.0, Chicago, Illinois, United States) and R software (version 4.0.5, Vienna, Austria).

## Results

### Population Characteristics

A total of 3,142 patients who underwent CAG or PCI were eventually enrolled in this study. Among them, 483 (15.4%) patients were diagnosed with CI-AKI after contrast exposure. Table 1 presented the descriptive data of baseline characteristics. Compared with patients without CI-AKI, patients with CI-AKI were significantly older (69.6 ± 11.2 years vs. 66.4 ± 10.7 years, *p* < 0.001), less number of males (59.2% vs. 68.3%, *p* < 0.001), more diabetic (35.4% vs. 26.1%, *p* < 0.001), and more hypertensive (70.2% vs. 64.6%, *p* = 0.021). Moreover, patients with CI-AKI had higher levels of C-reactive protein (CRP) [4.2 (1.4, 16.1) mg/L vs. 1.9 (0.8, 6.2) mg/L, *p* < 0.001], N-terminal pro-B-type natriuretic peptide (NT-proBNP) [646.0 (480.0, 3,988.0) pg/ml vs. 463.0 (121.0, 1528.0) pg/ml, *p* < 0.001], FBG (8.0 ± 3.7 mmol/L vs. 6.8 ± 2.8 mmol/L, *p* < 0.001), and HbA1c (6.7 ± 1.6% vs. 6.4 ± 1.3%, *p* < 0.001) and a lower level of eGFR [75.1 ± 28.6 ml/(min×1.73 m^2^) vs. 79.2 ± 22.1 ml/(min×1.73 m^2^), *p* < 0.001]. Moreover, patients with CI-AKI tended to be exposed to higher doses of the contrast medium [100.0 (60.0, 150.0) mg vs. 80.0 (50.0, 140.0) mg, *p* = 0.001]. However, there were no significant differences in total cholesterol (TC) and low-density lipoprotein cholesterol (LDL-C) between the groups (*p* for all>0.05).

**TABLE 1:** Descriptive statistics for baseline characteristics of patients with/without CI-AKI.

Characteristics	Overall (*n* = 3,142)	Contrast-induced acute kidney injury		*p* value
		No (*n* = 2659)	Yes (*n* = 483)	
Demographic features				
Age, years old	66.9 ± 10.8	66.4 ± 10.7	69.6 ± 11.2	<0.001*
Male, n (%)	2103 (66.9)	1817 (68.3)	286 (59.2)	<0.001*
BMI, kg/m^2^	24.5 ± 5.4	24.4 ± 5.4	25.2 ± 5.5	0.022*
Diabetes, n (%)	865 (27.5)	694 (26.1)	171 (35.4)	<0.001*
Hypertension, n (%)	2058 (65.5)	1719 (64.6)	339 (70.2)	0.021*
Laboratory data				
FBG, mmol/L	7.0 ± 3.0	6.8 ± 2.8	8.0 ± 3.7	<0.001*
HbA1c, %	6.5 ± 1.4	6.4 ± 1.3	6.7 ± 1.6	<0.001*
HGI	0 ± 1.16	0 ± 1.09	0 ± 1.45	0.939
SCr on admission, umol/L	77.0 (65.0, 95.0)	77.0 (65.5, 94.0)	74.0 (61.0, 102.5)	0.205
Proportion of SCr elevation, %	3.8 (−4.6, 16.1)	1.4 (−6.1, 9.1)	41.0 (31.4, 58.8)	<0.001*
eGFR, ml/(min×1.73 m^2^)	78.5 ± 23.2	79.2 ± 22.1	75.1 ± 28.6	<0.001*
TC, mmol/L	4.13 ± 1.20	4.13 ± 1.19	4.12 ± 1.22	0.752
LDL-C, mmol/L	2.22 ± 0.92	2.22 ± 0.92	2.26 ± 0.94	0.358
NT-proBNP, pg/ml	574.9 (141.0, 1911.0)	463.0 (121.0, 1528.0)	646.0 (480.0, 3,988.0)	<0.001*
CRP, mg/L	2.2 (0.9, 7.0)	1.9 (0.8, 6.2)	4.2 (1.4, 16.1)	<0.001*
PCI procedure data				
CTO, n (%)	160 (5.1)	137 (5.2)	23 (4.8)	0.532
Lesion location, n (%)				
LM	104 (3.3)	86 (3.2)	18 (3.7)	0.662
LAD	743 (23.6)	613 (23.1)	130 (26.9)	0.175
LCX	269 (8.6)	220 (8.3)	49 (10.1)	0.234
RCA	373 (11.9)	310 (11.7)	63 (13.0)	0.669
Total length of stents, mm	40.0 (28.0, 66.0)	42.0 (28.0, 66.0)	38.0 (27.8, 60.0)	0.472
Volume of the contrast agent, mg	80.0 (50.0, 150.0)	80.0 (50.0, 140.0)	100.0 (60.0, 150.0)	0.001*
Type of the contrast agent, n (%)				
Isotonic	1106 (35.2)	935 (35.2)	171 (35.4)	0.919
Hypotonic	2036 (64.8)	1724 (64.8)	312 (64.6)	0.919
Medication, n(%)				
Statin	2603 (82.8)	2215 (83.3)	388 (80.3)	0.127
Beta blocker	1577 (50.2)	1311 (49.3)	266 (55.1)	0.022*
ACEI or ARB	1513 (48.2)	1288 (48.4)	225 (46.6)	0.483
Furosemide injection	490 (15.6)	327 (12.3)	163 (33.7)	<0.001*

Continuous variables are presented as mean ± standard deviation or median (interquartile range) according to distribution, and categorical variables are presented as numbers and proportions. ACEI indicates angiotensin-converting enzyme inhibitor; ARB, angiotensin receptor antagonist; BMI, body mass index; CI-AKI, contrast-induced acute kidney injury; CRP, C-reactive protein; CTO, chronic total occlusion; eGFR, estimated glomerular filtration rate; FBG, fasting blood glucose; HbA1c, glycated hemoglobin; HGI, hemoglobin glycation index; LAD, left anterior descending artery; LCX, left circumflex artery; LDL-C, low-density lipoprotein cholesterol; LM, left main coronary artery; NT-proBNP, N-terminal pro-B-type natriuretic peptide; RCA, right coronary artery; SCr, serum creatinine; TC, total cholesterol. *p < 0.05.

### Association of the HGI With Proportion of SCr Elevation

As shown in Table 2, the β coefficient with 95% confidence interval was calculated on both positive and negative sides of the HGI (HGI <0 and HGI ≥0). The multivariable linear regression analysis after full adjustment showed a strongly negative association of the HGI with the proportion of SCr elevation when HGI <0 [*β* = -9.537, 95% CI (−12.057 to −7.017), *p* < 0.001], whereas a slightly positive association was noted when HGI ≥0 [*β* = 1.655, 95% CI (0.125 to 3.186), *p* = 0.034].

**TABLE 2:** Linear regression analysis of the HGI with the proportion of SCr elevation.

	Model 1		Model 2		Model 3	
	β coefficient (95% CI)	*p* value	β coefficient (95% CI)	*p* value	β coefficient (95% CI)	*p* value
HGI <0	−10.078 (−12.662 to −7.494)	<0.001*	−10.284 (−12.858 to −7.709)	<0.001*	−9.537 (−12.057 to -7.017)	<0.001*
HGI ≥0	1.578 (0.016 to 3.141)	0.048*	1.671 (0.104 to 3.238)	0.037*	1.655 (0.125 to 3.186)	0.034*

Model 1 adjusted for none.

Model 2 adjusted for age (years old), gender (male or female), hypertension (yes or no) and eGFR (ml/min × 1.73 m^2^).

Model 3 additionally adjusted for exposure volume of the contrast agent (mg), administration of statin (yes or no), administration of ACEI/ARB (yes or no), and furosemide injection (yes or no).

ACEI, indicates angiotensin-converting enzyme inhibitor; ARB, angiotensin receptor antagonist; CI, confidence interval; eGFR, estimated glomerular filtration rate; HGI, hemoglobin glycation index; SCr, serum creatinine. *p < 0.05.

### Association of the HGI With CI-AKI

**FIGURE 3:**
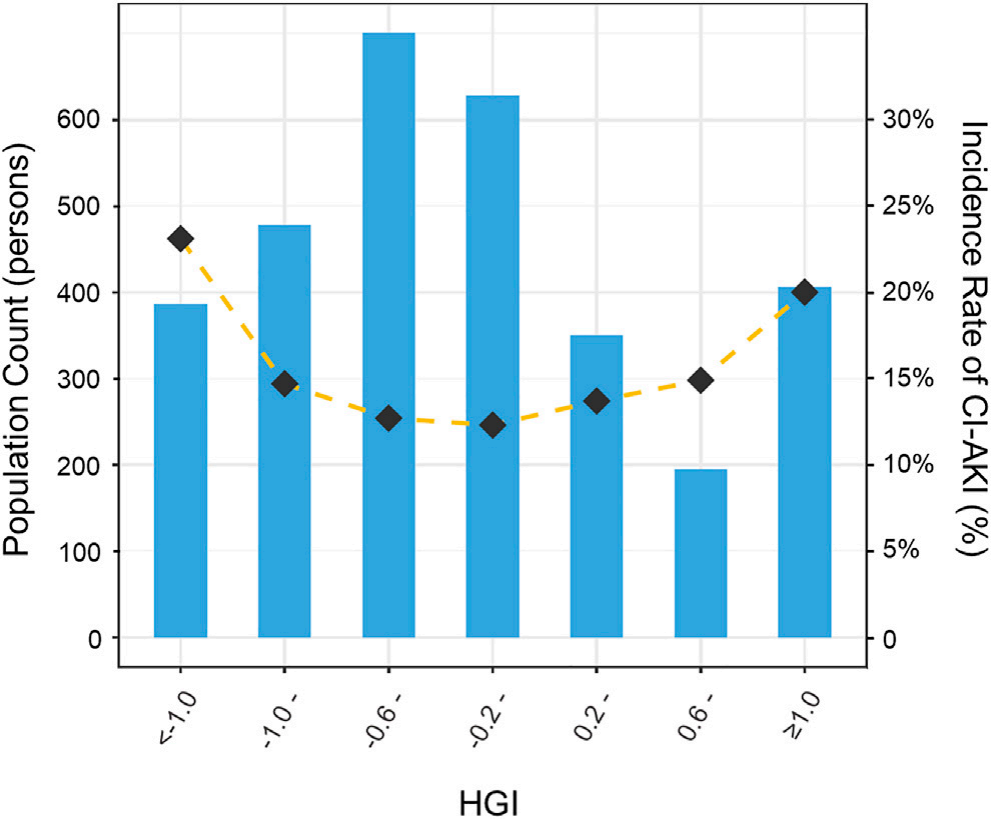
Distribution of population and the incidence of CI-AKI according to HGI categories. Bar plots showed the distribution of the population with different levels of HGI. Gold dashed line indicated the changing trend in the incidence of CI-AKI with different HGI categories. HGI indicates hemoglobin glycation index; CI-AKI, contrast-induced acute kidney injury.

**FIGURE 4:**
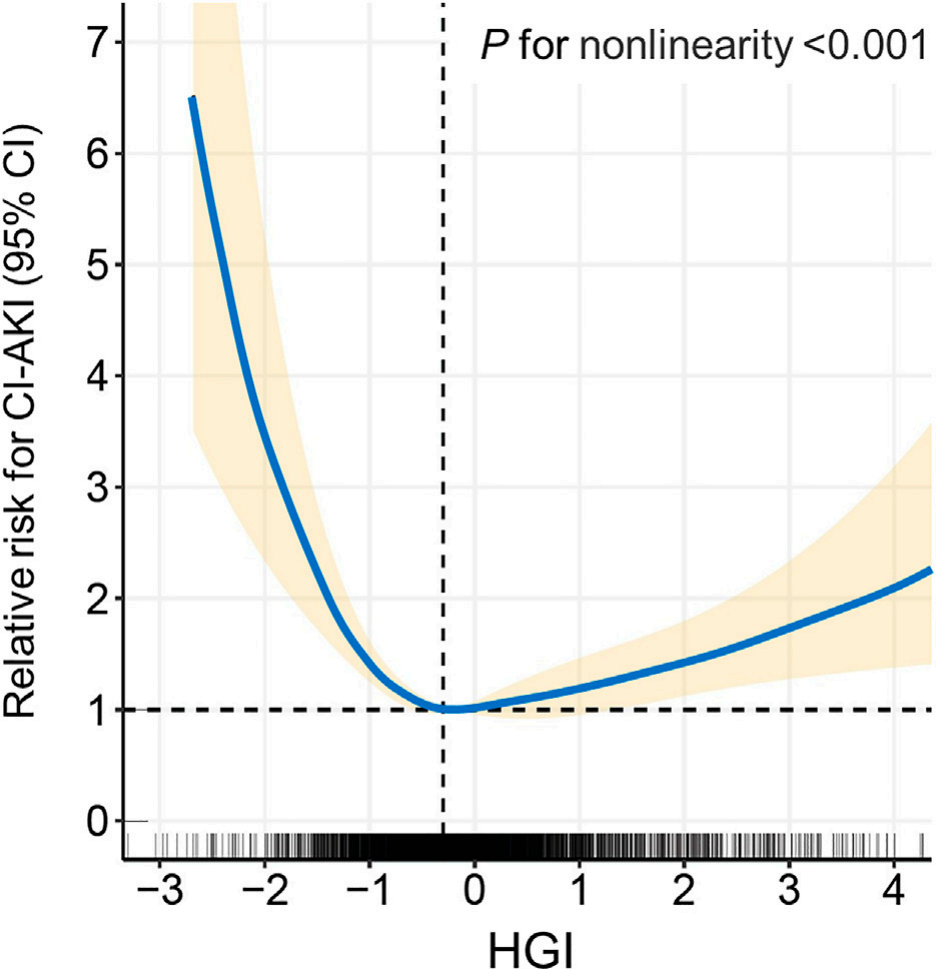
Restricted cubic spline (RCS) analysis of the HGI with the incidence of CI-AKI. The spline plot showed the relative risk for CI-AKI according to HGI levels on a continuous scale. Four knots were chosen for coding HGI with the restricted cubic spline function. HGI indicates hemoglobin glycation index; CI-AKI, contrast-induced acute kidney injury.

All patients were divided into seven distinct groups at equal interval according to the HGI value. The curve in Figure 3 indicated that the population with a higher absolute value of the HGI had a higher incidence of CI-AKI. In Figure 4, a significantly nonlinear association between the HGI and the incidence of CI-AKI was examined and demonstrated by the RCS model (*p* for non-linearity <0.001). Apparent reduction in the risk of CI-AKI can be observed in the spline plot when the HGI gradually reduced to 0.

Next, the association between the HGI and CI-AKI was evaluated and modeled using modified Poisson’s regression analysis when treating the fourth category (−0.2 ≤ HGI <0.2) as reference (Table 3). After multivariable adjustment for potential confounders, the highest and lowest level of HGI categories were proven to be strongly associated with CI-AKI [(<−1.0 vs. −0.2 to 0.2): aRR = 1.897, 95% CI (1.467 to 2.452), *p* < 0.001 (≥1.0 vs. −0.2 to 0.2): aRR = 1.545, 95% CI (1.171 to 2.037), *p* = 0.002].

**TABLE 3:** Modified Poisson’s regression analysis of HGI categories with CI-AKI.

HGI	Cases/overall (%)	Model 1		Model 2		Model 3	
		aRR (95%CI)	*p* value	aRR (95%CI)	*p* value	aRR (95%CI)	*p* value
<-1.0	89/386 (23.1)	1.880 (1.425 to 2.482)	<0.001*	1.973 (1.506 to 2.585)	<0.001*	1.897 (1.467 to 2.452)	<0.001*
≥-1.0, <-0.6	70/477 (14.7)	1.197 (0.886 to 1.617)	0.242	1.286 (0.959 to 1.725)	0.093	1.319 (0.988 to 1.760)	0.060
≥-0.6, <-0.2	89/700 (12.7)	1.037 (0.780 to 1.379)	0.803	1.085 [0.822 to 1.432]	0.564	1.127 (0.858 to 1.479)	0.390
≥-0.2, <0.2	77/628 (12.3)	1 (Reference)		1 (Reference)		1 (Reference)	
≥0.2, <0.6	48/350 (13.7)	1.119 (0.799 to 1.565)	0.513	1.095 (0.787 to 1.523)	0.590	1.152 (0.835 to 1.589)	0.389
≥0.6, <1.0	29/195 (14.9)	1.213 (0.817 to 1.802)	0.339	1.141 (0.766 to 1.699)	0.517	1.207 (0.815 to 1.787)	0.348
≥1.0	81/406 (20.0)	1.627 (1.223 to 2.166)	0.001*	1.617 (1.216 to 2.151)	0.001*	1.545 (1.171 to 2.037)	0.002*

Model 1 adjusted for none.

Model 2 adjusted for age (<70 or ≥70 years), gender (male or female), hypertension (yes or no), and eGFR (<30, 30–59, 60–89, ≥90 ml/min×1.73 m^2^).

Model 3 additionally adjusted for exposure volume of the contrast agent (<60, 60–119, ≥120 mg), administration of statin (yes or no), administration of ACEI/ARB (yes or no), and furosemide injection (yes or no).

ACEI, indicates angiotensin-converting enzyme inhibitor; ARB, angiotensin receptor antagonist; aRR, adjusted relative risk; CI, confidence interval; CI-AKI, contrast-induced acute kidney injury; eGFR, estimated glomerular filtration rate; HGI, hemoglobin glycation index. *p < 0.05.

### Exploratory Analysis

The consistency of the association between the HGI and the incidence of CI-AKI was assessed according to the stratification of HbA1c levels (Table 4). Exploratory analysis revealed that the highest and lowest level of the HGI were still tightly associated with higher incidence of CI-AKI in the sub-populations (HbA1c <6% or ≥6% population), which was in great agreement with the main results in the whole population.

**TABLE 4:** Exploratory analysis according to stratification of HbA1c levels.

Subgroups	HGI	Cases/overall (%)	aRR (95% CI)	*p* value
HbA1c <6%	<−1.0	67/299 (22.4)	2.406 (1.530 to 3.785)	<0.001*
204/1431 (14.3%)	≥-1.0, <-0.6	55/400 (13.8)	1.574 (0.983 to 2.523)	0.059
	≥-0.6, <-0.2	61/517 (11.8)	1.338 (0.837 to 2.137)	0.224
	≥-0.2, <0.2	19/211 (9.0)	1 (Reference)	
	≥0.2, <0.6	2/4 (50%)	2.954 (1.427 to 6.113)	0.004*
	≥0.6, <1.0	*NA*	*NA*	*NA*
	≥1.0	*NA*	*NA*	*NA*
HbA1c ≥6%	<-1.0	22/87 (25.3)	1.814 (1.226 to 2.684)	0.003*
279/1711 (16.3%)	≥-1.0, <-0.6	15/77 (19.5)	1.491 (0.898 to 2.473)	0.122
	≥-0.6, <-0.2	28/183 (15.3)	1.223 (0.819 to 1.826)	0.325
	≥-0.2, <0.2	58/417 (13.9)	1 (Reference)	
	≥0.2, <0.6	46/346 (13.3)	1.003 (0.712 to 1.413)	0.988
	≥0.6, <1.0	29/195 (14.9)	1.092 (0.729 to 1.636)	0.671
	≥1.0	81/406 (20.0)	1.393 (1.040 to 1.866)	0.026*

aRR, indicates adjusted relative risk; CI, confidence interval; HbA1c, glycated hemoglobin; HGI, hemoglobin glycation index; NA, not available. *p < 0.05.

## Discussion

In this study, a high absolute value of the HGI (<−1.0 or ≥1.0) was proven to be strongly associated with CI-AKI in patients who underwent CAG or PCI. Compared to the positive value of the HGI, the negative value of the HGI was observed to increase the risk of CI-AKI more obviously. In addition, in exploratory analysis, the abovementioned association remained consistent even in patients whose baseline HbA1c level was less than 6.0%.

Renal dysfunction has been shown to be associated with impaired glucose metabolism, and diabetes has already been demonstrated to be an independent risk factor for the occurrence of CI-AKI (Calvin et al., 2010; Succurro et al., 2010). HbA1c is extensively used to reflect the average blood glucose level in the short term to evaluate the patient’s glycemic control status and has been recommended as the diagnostic indicator of diabetes and prediabetes according to the International Expert Committee (The International Expert Committee, 2009). However, Herman et al. has found that HbA1c can be affected by a variety of factors and there remain considerable interindividual variations in HbA1c (Herman et al., 2007). To quantify the interindividual variation of HbA1c, HGI, a novel indicator of diabetes risk, was used in this study (Hempe et al., 2002). The HGI was calculated as the difference between the observed HbA1c and the predicted HbA1c which was estimated from the linear regression formula between HbA1c and FPG (Hsia et al., 2020). In the Action to Control Cardiovascular Risk in Diabetes (ACCORD) trial, diabetic patients with a high HGI exhibited increased risk for retinopathy and nephropathy at baseline (Hempe et al., 2015). Fiorentino et al. also reported that HGI was an independent risk factor of kidney dysfunction in White non-diabetic individuals (Fiorentino et al., 2017b). Compared to previous studies that mainly grouped HGI according to tertiles or quartiles, this large sample study not only performed more nuanced and specific classifications but also took the positive and negative value of HGI into account. The current study demonstrated that a high absolute value of HGI was strongly associated with CI-AKI in patients undergoing CAG or PCI, indicating that perioperative glycemic control may be a potential strategy for preventing CI-AKI even in patients with good glycemic control.

Although the pathophysiology of CI-AKI remains complex and unclear, the relationship between high HGI and CI-AKI can be explained by the following plausible mechanisms (Vlachopanos et al., 2019). First, the HGI reflects the propensity of nonenzymatic glycation processes. Higher HGI was associated with the increased formation of advanced glycation end products (AGEs) (Kim et al., 2018). The AGEs can induce oxidative stress, pro-fibrotic responses, and endothelial dysfunction, which may result in the development of tubular and glomerular injury, thus increasing the risk of CI-AKI (Wendt et al., 2003; Busch et al., 2010). Second, inflammation plays a key role in the incidence and development of CI-AKI (Kwasa et al., 2014). Also, the correlation between elevated HGI and increased inflammatory status has been proven to be significantly strong (Liu et al., 2015). Therefore, inflammation may be another mechanistic factor linking HGI to CI-AKI. As the current study demonstrated, patients who suffered CI-AKI had higher levels of CRP. Third, higher HGI was associated with individual metabolic disorders, including glucose metabolism, protein metabolism, and lipid metabolism (Marini et al., 2017). Renal metabolic disorders inevitably increase the burden on the kidney and aggravate renal pathological changes, which ultimately lead to CI-AKI (Yoon et al., 2020). Similar results were also demonstrated in this study: patients with CI-AKI had a lower pre-procedure eGFR level (75.1 ± 28.6 vs. 79.2 ± 22.1 ml/(min×1.73 m^2^), *p* < 0.001), indicating poorer renal function. Fourth, kidney angiosclerosis and glomerulosclerosis alter the blood and hemodynamics of kidney tissues (Glassock and Rule, 2016). Nagayama et al. has demonstrated that a high HGI could promote the development of systemic arterial stiffening, including nephroangiosclerosis, which may increase the incidence of CI-AKI subsequently (Nagayama et al., 2020).

While studies on how the negative value of HGI facilitates the occurrence of CI-AKI are still limited, this problem may be explained reasonably by some speculations. It is well-known that HbA1c cannot reflect the real-time fluctuations in blood glucose. In pre-procedural patients with a lower HGI who should have higher FBG levels, lower HGI may demonstrate poorer control of blood glucose recently, regardless of the insufficient dose of hypoglycemic agents in diabetes patients or the changes of lifestyle or diet in non-diabetes patients. High-glucose status enhances the formation of reactive oxygen species and aggravates renal parenchymal hypoxia (Heyman et al., 2010). Reactive oxygen species accumulation and renal parenchymal hypoxia are mutually influenced and promoted and eventually lead to CI-AKI.

In spite of significant findings being mentioned, the present study still has some limitations. First, as a retrospective study, the inherent bias in patient selection was inevitable. Therefore, scientifically designed prospective studies could help provide more value. Second, temporary and short‐term glucose-lowering treatment may lead to fluctuations of HGI, so this study excluded the patients who initiated or adjusted the hypoglycemic regimen within 3 months. Third, the variability of the HGI in different types or degrees of myocardial infarction was unavoidable. Further research studies in diverse sub-populations are needed. Fourth, there are marked ethnic differences in HbA1c levels and the results of this study only based on Chinese individuals. Therefore, whether the findings could be extended to other ethnic groups remains unclear.

## Conclusion

A high absolute value of the HGI was closely associated with a higher incidence of CI-AKI in patients undergoing CAG or PCI.

## Data Availability

The original contributions presented in the study are included in the article/Supplementary Material, further inquiries can be directed to the corresponding authors.

## Abbreviations

ACCORD, Action to Control Cardiovascular Risk in Diabetes; ACEI, angiotensin-converting enzyme inhibitor; AGEs, advanced glycation-end products; ARB, angiotensin receptor antagonist; aRR, adjusted relative risk; BMI, body mass index; CAD, coronary artery disease; CAG, coronary angiography; CI-AKI, contrast-induced acute kidney injury; CRP, C-reactive protein; eGFR, estimated glomerular filtration rate; ESUR, European Society of Urogenital Radiology; FBG, fasting blood glucose; HbA1c, glycated hemoglobin; HGI, hemoglobin glycation index; HIS, Hospital Information System; LDL-C, low-density lipoprotein cholesterol; LVEF, left ventricular ejection fraction; NOS, Newcastle–Ottawa Scale; NT-proBNP, N-terminal pro-B-type natriuretic peptide; NYHA, New York Heart Association; PCI, percutaneous coronary intervention; RCS, restricted cubic spline; SCr, serum creatinine; SD, standard deviation; STROBE, Strengthening the Reporting of Observational Studies in Epidemiology; TC, total cholesterol.
